# Dr. Charles G. Combs

**Published:** 1913-08

**Authors:** 


Dr. Charles G. Combs (not listed in State Directory) died July 17 at his home in Rochester, aged 52.

